# Evidence of antibacterial properties by endophytic fungi of Hazelnut (*Corylus avellana*)

**DOI:** 10.3389/fmicb.2026.1783646

**Published:** 2026-03-05

**Authors:** Rosario Nicoletti, Elvira Ferrara, Andrea Becchimanzi, Beata Zimowska, Milena Petriccione

**Affiliations:** 1 Council for Agricultural Research and Economics, Research Center for Olive, Fruit and Citrus Crops, Caserta, Italy; 2 Department of Agricultural Sciences, University of Naples Federico II, Portici, Italy; 3 Department of Plant Protection, Subdepartment of Phytopathology and Mycology, University of Life Sciences, Lublin, Poland

**Keywords:** antibiosis, defensive mutualism, hazelnut mycobiome, pathogenic bacteria, phase-out of copper products

## Abstract

New control strategies are necessary for the treatment of the bacterial diseases of hazelnut (*Corylus avellana*) incited by *Pseudomonas avellanae* and *Xanthomonas arboricola* pv. *corylina*, following the programmed phasing out of copper-based anti-cryptogamics. Based on recent evidence gathered on many crops, endophytic fungi are credited for playing a role as defensive mutualists of plants. Thus, an investigation was carried out in the hazelnut growing areas in Southern Italy in the aim to identify endophytic fungi possessing antimicrobial properties against these two pathogens. A panel of 50 endophytic isolates was selected, including species which are already known as being part of the hazelnut mycobiome, along with a few new records. These isolates were tested for antibiosis in an *in vitro* assay consisting in the inoculation of the two bacterial pathogens in their culture filtrates. Four isolates, belonging to *Cladosporium perangustum, Talaromyces purpureogenus, Nemania diffusa* and the *Hypoxylon fuscum* species complex, displayed consistent inhibitory effects, inducing about 90% growth suppression of both bacteria. Their capacity to effectively act as biocontrol agents will be further tested *in planta*.

## 1 Introduction

Hazelnut (*Corylus avellana* L.) is a stone fruit crop with a relevant impact on the socio-economic conditions in several rural areas in Italy (Zinnanti et al., 2019; Vinci et al., 2023; Nera et al., 2020; Biagetti et al., 2023; Pergola et al., 2024). It is primarily valued for its kernels, which are consumed raw, roasted, or, more commonly, as ingredients in confectionery, baked goods, and various food products (Fuso et al., 2021; Romero-Aroca et al., 2021). In 2023, global hazelnut production was estimated at around 1.15 million tons; Turkey is the dominant producer of shelled hazelnuts, supplying about 56% of the world market, followed by Italy with a share of approximately 9% (FAOStatistics, 2025; Yildirim et al., 2024).

Hazelnut production is affected by various biotic adversities, which management requires the use of pesticides and fungicides. Among them, copper-based products are traditionally employed for the treatment of cryptogamic diseases; however, their phase out has been programmed in the European Union because of negative side effects on the environment (Nicoletti et al., 2022b; Tamm et al., 2022). Therefore, the need has arisen for the hazelnut growers to find alternative control measures in the management of bacterial diseases, such as canker caused by *Pseudomonas avellanae* and blight caused by *Xanthomonas arboricola* pv. *corylina*, for which no other effective chemicals are currently available. Since the early 2000s, these bacteria have been reported to affect hazelnut orchards in Italy, Poland and other European countries (Krol et al., 2004; Lamichhane and Varvaro, 2014; Nicoletti et al., 2022b). In Central Italy, where hazelnut cultivation exceeds 20,000 ha, *P. avellanae* caused severe canker outbreaks on approximately 1,000 ha, and more than 40,000 trees were lost (Scortichini et al., 2002a). On the other hand, *X. arboricola* pv. *corylina* is a major limiting factor in nurseries, causing significant mortality in young orchards (less than four years old), where losses can be higher than 10%; in older orchards plant death rarely occurs, but branch dieback leads to reduced yield (Pulawska et al., 2010).

In the context of a rising awareness that the use of chemicals in agriculture must be reduced, the phase out of copper products offers an opportunity to explore eco-friendly alternatives for the management of these hazelnut pathogens. Recently, the role of plant microbiota has been increasingly considered for its possible exploitation in plant protection, particularly with reference to tree crops (Rabiey et al., 2019; Pereira et al., 2023). Indeed, the antimicrobial properties expressed by many symbiont microbes are credited as a major factor enabling fruit trees to escape or minimize the aggression by latent pathogens which unevenly infect plants growing in conducive climatic and/or cultivation conditions, as is the case of the above-mentioned bacteria. On the other hand, interactions in the plant microbiome may occasionally result in a support for disease agents (Busby et al., 2016). Data concerning the endophytic associates of hazelnut and their dual role in plant protection are currently limited (Nicoletti and Zimowska, 2023), making investigations in the field fundamental in view of possible exploitation in the management of these pathogens. This paper reports the results of an investigation on the *in vitro* antibacterial aptitude of a panel of endophytic fungi recovered from asymptomatic hazelnut plants in Southern Italy, with the aim to select isolates which can be further examined for their biocontrol potential and the production of bioactive compounds.

## 2 Materials and methods

### 2.1 . Isolation of endophytic fungi

Isolation of endophytic fungi was carried out during the spring of 2024 from asymptomatic plants from both cultivated orchards (Altavilla Irpina, Caserta, Cicala di Nola, Roccarainola, Serino, Summonte, Taverna Figura, Tricarico, Tufo) or wild contexts (Astroni Nature Reserve, Apice, Mercogliano, Pratola Serra, Venticano) in Southern Italy. Segments of secondary branches (about 5 cm in length) were cut and brought to the laboratory in sterile plastic bags. They were disinfected by immersion in 70% ethanol for 1 min, followed by immersion in 4% sodium hypochlorite for 3 min, and rinsing three times in sterile distilled water. After removing the bark with a scalpel, longitudinal sections of about 1 cm were cut and placed on potato dextrose agar (PDA: Oxoid, Basingstoke, United Kindgom) amended with 85% lactic acid (1 mL L^−1^) in 9-cm Petri dishes. The dishes were incubated in darkness at 25 °C for up to two weeks and inspected daily. Hyphal tips of the emerging fungal colonies were transferred in pure cultures into fresh PDA dishes, and stored at 4 °C. In total, a sample of 120 isolates was collected, from which 50 strains were randomly selected to be used for the subsequent steps. All the isolates are stored in the microbial collection of Research Center for Olive, Fruit and Citrus Crops by Council for Agricultural Research and Economics in Caserta, Italy.

### 2.2 . Antibacterial assays

For the assessment of their antibacterial properties, liquid cultures (10 mL) of each of the selected strains were prepared in potato dextrose broth (PDB, Oxoid) in sterile glass tubes, which were inoculated with a mycelial plug from actively growing cultures on PDA of each isolate and incubated in darkness at 25 °C. After two weeks, the cultures were filtered through 0.45 μm cellulose-acetate Minisart filters (Sartorius, Göttingen, Germany), and the filtrates (4.5 mL) put into sterile borosilicate glass tubes (10 mL). An original antibacterial assay was carried out by directly inoculating the tubes with two bacterial strains from our collection, namely CRAFRUEC1 of *P. avellanae* (PA) (Scortichini et al., 2013) and ISF Nc 18 of *X. arboricola* pv. *corylina* (XAC) (Scortichini et al., 2002b), after having preliminarily checked their capacity to grow in PDB. More in detail, each culture filtrate was added with 0.5 mL of a suspension, adjusted at 1–2 × 10^3^ cfu mL^−1^ using the standard plate count method, of the bacterial strains grown on nutrient agar (Oxoid) added with 3% sucrose (NSA) for 48 h at 25 ± 1 °C. Optical density at 600 nm (OD600) was measured through a UV-VIS spectrometer (Jasco V-530, Milan, Italy) to monitor bacterial growth in liquid culture over a 5-day incubation period at 25 ± 1 °C. The assays were carried out in triplicate. Based on measurements of the suspension turbidities, the percentage of growth inhibition (negative values) or promotion (positive values) were estimated by comparing the OD600 values of the samples treated with the filtrate with those of the control cultures grown in PDB only.

### 2.3 Statistical analysis

All the resulting values are expressed as mean ± standard deviation (SD) of three independent replicates (*n* = 3). The significance of the mean differences was analyzed by means of ANOVA and the Duncan's test at 5% level of significance, which were carried out using SPSS v.20.0 statistical software (IBM Corporation, Armonk, NY, USA).

### 2.4 . Identification of selected fungal strains

Mycelia harvested from the liquid cultures were subjected to DNA extraction using the Quick-DNA Fungal/Bacterial Kit (Zymo Research, Irvine, CA, USA) in accordance with the manufacturer's protocol. The DNA extracted from the mycelia was quantified spectrophotometrically, and its purity was assessed by measuring the absorbance at 260 nm and 280 nm. Preliminary taxonomic identification was based on ITS1 (5′TCC GTA GGT GAA CCT GCG G 3′) and ITS4 (5′TCC TCC GCT TAT TGA TAT GC 3) sequencing (White et al., 1990). PCR amplification was carried out in 20 μL of PCR reaction mixture, containing 25 ng DNA, 0.5 U Taq-DNA polymerase (AmpliTaq Gold, Applied Biosystems Inc., Foster City, CA, USA), 1X PCR buffer, 2 mM MgCl_2_, 200 mM dNTPs and 0.5 mM of each primer (Thermo Fisher, Waltham, MA, USA). Reactions involved one cycle at 94 °C for 5 min, followed by 40 cycles with a denaturation step at 94 °C for 40 s, an annealing step at 55 °C for 45 s, and an extension step at 72 °C for 90 s, followed by one cycle at 72 °C for 7 min. Aliquots (10 μL) of each PCR product were separated by electrophoresis on 1% agarose gel prepared in 1X Tris-acetate-EDTA buffer and visualized under UV light after staining with Gel Doc XR+ (Biorad, Hercules, CA, USA). To enhance the accuracy of species-level identification, additional molecular characterization was carried out for the isolates which better performed in the antibacterial assays, using the RNA polymerase second largest subunit (*rpb2*), β-tubulin (*benA*) and translation elongation factor 1-α (*tef* ) gene regions as complementary phylogenetic markers. PCR amplification was performed using the primer pairs RPB2-5F/RPB2-7cR, T11/T22 (for isolate HN15E), Bt1a/Bt1b (for isolate HN15G), Bt2a/Bt2b (for isolate HN10I), and EF1-728F/EF1-986R (for isolate HN01C), with reaction conditions optimized for each target locus (Liu et al., 1999; Glass and Donaldson, 1995; Guevara-Suarez et al., 2016; Nguyen et al., 2021; Pi et al., 2021; Vocadlova et al., 2023). Molecular weight markers were included in each run (GeneRuler 100-bp DNA Ladder Plus; MBI Fermentas, Thermo Fisher). Amplified DNA from PCR was sequenced by Eurofins service (Eurofins Genomics Europe Pharma and Diagnostics Products & Services, Cologne, Germany). All the DNA marker sequences obtained in this study have been deposited at the NCBI GenBank database and are available for use by third parties with the codes listed in Table 1, where the closest taxonomic match determined through BLAST is also reported. In addition, ITS sequences were used for representing the phylogenetic relationships within the set of endophytic isolates through a dendrogram obtained following the method described in a recent paper (Becchimanzi et al., 2025).

**Table 1:** Endophytic fungal isolates from hazelnut trees used for antibacterial assays.

**Isolate**	**Location**	**ITS**	**Closest GenBank match°**	**Identification**
HN01A	Astroni Nature Reserve	PP683249	*Biscogniauxiamediterranea* ^99^	*B. mediterranea*
HN01C	Astroni Nature Reserve	PP683251	*Cladosporiumperangustum* ^100^	*C*.perangustum^*^
HN01E	Astroni Nature Reserve	PP683253	*Melanconiumhedericola* ^99^	*M. hedericola*
HN02B	Caserta	PX601487	*Diaporthefoeniculina* ^100^	*Diaporthe* sp.
HN02H	Caserta	PX601498	*Angustimassarina* sp. ^100^	*Angustimassarina* sp.
HN03A	Tufo	PX601507	*Alternariatenuissima* ^100^	*Alternaria* sp.
HN03D	Tufo	PX601468	*Nothophomaquercina* ^99^	Didymellaceae sp.
HN04A	Pratola Serra	PX601484	*B*.*mediterranea*^100^	*B. mediterranea*
HN05A	Taverna Figura	PX601471	*Cladosporiumcladosporioides* ^100^	*C. cladosporioides* s.c.
HN05B	Taverna Figura	PX601512	*B*.*mediterranea*^100^	*B. mediterranea*
HN06A	Venticano	PX601513	*Heteroxylariaoxyacanthae* ^99.5^	*H. oxyacanthae*
HN06D	Venticano	PX601511	*Phlebiaacerina* ^100^	*P. acerina*
HN07A	Apice	PX601486	*B*.*mediterranea*^99^	*B. mediterranea*
HN09B	Serino	PX601506	*Stemphylium* sp. ^100^	*Stemphylium* sp.
HN09C	Serino	PX601469	*P*.*acerina*^100^	*P. acerina*
HN10D	Tricarico	PX601499	*Alternariaalternata* ^99.8^	*Alternaria* sp.
HN10F	Tricarico	PX601496	*Cytospora* sp. ^99.5^	*Cytospora* sp.
HN10G	Tricarico	PX601470	*Angustimassarina* sp. ^98^	*Angustimassarina* sp.
HN10H	Tricarico	PX601465	*Cytosporaprunicola* ^99.5^	*Cytospora* sp.
HN10I	Tricarico	PX601494	*Talaromycespinophilus* ^100^	*Talaromycespurpureogenus* ^*^
HN10L	Tricarico	PX601502	*Cytospora* sp. ^99.6^	*Cytospora* sp.
HN10M	Tricarico	PX601501	*Trametesversicolor* ^100^	*T. versicolor*
HN11A	Tricarico	PX601503	*Alternaria* sp. ^100^	*Alternaria* sp.
HN11M	Tricarico	PX601510	Didymellaceae sp. ^100^	Didymellaceae sp.
HN11N	Tricarico	PX601472	*Alternariainfectoria* ^100^	*Alternaria* sp.
HN12O	Tricarico	PX601474	*Angustimassarina* sp. ^99^	*Angustimassarina* sp.
HN13A	Roccarainola	PX601500	*Cylindrobasidium* sp. ^99^	*Cylindrobasidium* sp.
HN13D	Roccarainola	PX601509	*Alternaria* sp. ^100^	*Alternaria* sp.
HN13F	Roccarainola	PX601480	*Diaporthe* sp. ^100^	*Diaporthe* sp.
HN13G	Roccarainola	PX601497	*D*.*foeniculina*^100^	*D. foeniculina*
HN13H	Roccarainola	PX601473	*Diaportherudis* ^100^	*D. rudis*
HN13M	Roccarainola	PX601467	*D*.*foeniculina*^100^	*D. foeniculina*
HN14C	Cicala di Nola	PX601475	*Paraconiothyriumbrasiliense* ^99^	*P. brasiliense*
HN14D	Cicala di Nola	PX601485	*Alternaria* sp. ^99^	*Alternaria* sp.
HN14G	Cicala di Nola	PX601504	*D*.*foeniculina*^100^	*D. foeniculina*
HN14I	Cicala di Nola	PX601466	*Angustimassarina* sp. ^100^	*Angustimassarina* sp.
HN14N	Cicala di Nola	PX601505	*Diaportheeres* ^99^	*D. eres*
HN15A	Altavilla Irpina	PX601492	Didymellaceae sp. ^99.43^	Didymellaceae sp.
HN15B	Altavilla Irpina	PX601483	*Hypoxylonfuscum* ^100^	*H. fuscum* s.c.
HN15D	Altavilla Irpina	PX601489	*D*.*foeniculina*^99^	*D. foeniculina*
HN15E	Altavilla Irpina	PX601479	*Nemaniadiffusa* ^99^	*N*.diffusa^*^
HN15F	Altavilla Irpina	PX601476	*H*.*fuscum*^98.85^	*H. fuscum* s.c.
HN15G	Altavilla Irpina	PX601495	*H*.*fuscum*^100^	*H. fuscum* s.c.^*^
HN15H	Altavilla Irpina	PX601490	*Hypoxylonhoweanum* ^100^	*H. howeanum*
HN15I	Altavilla Irpina	PX601488	*H*.*fuscum*^100^	*H. fuscum* s.c.
HN16B	Mercogliano	PX601477	*Alternaria* sp. ^100^	*Alternaria* sp.
HN16D	Mercogliano	PX601493	Didymellaceae sp. ^100^	Didymellaceae sp.
HN16E	Mercogliano	PX601481	Pleosporales sp. ^100^	Pleosporales sp.
HN17A	Summonte	PX601482	*P*.*brasiliense*^100^	*P. brasiliense*
HN17B	Summonte	PX601491	*B*.*mediterranea*^99^	*B. mediterranea*

°*GenBank blasts yielded scores between 98 and 100%, as indicated next to the taxon name*. ^*^ *Identification of these isolates was confirmed through sequencing additional markers; namely, tef for HN01C (PP740379), rpb2 and benA for HN10I (PX619231, PX619234), HN15E (PX619229, PX619232) and HN15G (PX619230, PX619233). s.c.: species complex*.

## 3 Results

Data concerning location and taxonomic identification based on molecular markers of the 50 hazelnut endophytic isolates selected in our study are reported in Table 1. Most of them were identified as species already reported for their endophytic occurrence on hazelnut (Nicoletti and Zimowska, 2023). The only exceptions are the ligniculous basidiomycetes *Phlebia acerina* (Polyporales, Meruliaceae), *Trametes versicolor* (Polyporales, Polyporaceae) and *Cylindrobasidium* sp. (Agaricales, Physalacriaceae); however, even if representing new records as endophytes, these species are well known in association with forest trees, including hazelnut (Dogan et al., 2005; Tomsovský et al., 2006; Ordynets et al., 2017).

All the other isolates in our sample were determined to belong to the Ascomycetes. Among them, species in the Xylariales (Pezizomycotina, Sordariomycetes), which are on fire with reference to their capacity to synthesize bioactive secondary metabolites with possible ecological significance (Stadler et al., 2007; Becker and Stadler, 2021; Franco et al., 2022), appear to be dominant. Particularly, with five isolates from five different locations, *Biscogniauxia mediterranea* is the most frequent taxon. Its occurrence as an endophyte is known on many tree species (Nicoletti et al., 2020, 2021; Costa et al., 2022); nevertheless, it has also been reported from cankers in declining hazelnut plants (Varvaro et al., 2011), and its real ecological role on this tree deserves to be more carefully investigated. *Hypoxylon fuscum*, of which four isolates were recovered at the site of Altavilla Irpina along with an isolate of the distantly related *Hypoxylon howeanum*, is reported to be of frequent occurrence on hazelnut (Ma et al., 2022; Nicoletti and Zimowska, 2023). However, it was characterized as a species complex (s.c.) in a recent study (Lambert et al., 2021); thus, a more circumstantial identification of our strains, which denote some extent of genetic variation according to the GenBank blasts, requires the examination by specialists. The species *Nemania diffusa* was previously identified from hazelnut specimens in Bohemian herbaria (Zíbarová and Kout, 2017); it is also widespread as an endophytic associate of forest trees, and it has recently been reported to be possibly involved in defensive mutualism of ash (*Fraxinus excelsior*), based on production of cytochalasins (Demir et al., 2025). Finally, *Heteroxylaria oxyacanthae* (formerly *Xylaria oxyacanthae*) was not previously found as a hazelnut endophytic associate; however, it has been frequently recovered from fruits of a wide range of trees, in the context of a widespread geographical diffusion (Himani and Krishnappa, 2020; Kim et al., 2025).

Four isolates from four different locations could only be identified as belonging to the Didymellaceae (Dothideomycetes, Pleosporales). The closest GenBank match of one of them is *Nothophoma quercina*, a species already reported for endophytic occurrence on hazelnut in Iran (Kashanian et al., 2021). However, the results of blasting the corresponding ITS sequences were not univocal, so that we preferred to abstain from being more circumstantial in species ascription; also considering that this is one of the largest fungal families, still in course of further taxonomic assessments (Zimowska, 2021). Four isolates in another Pleosporales family (Amorosiaceae) from three different sites were determined to belong to the recently typified genus *Angustimassarina* (Thambugala et al., 2015). These fungi were also previously identified in the mentioned Iranian study (Kashanian et al., 2021); considering the remarkable geographical distance, their more widespread occurrence in association with hazelnut can be expected to be pointed out in the future. Quite interestingly, also *Paraconiothyrium brasiliense* (Pleosporales, Didymosphaeriaceae), recovered at two sites in our study, was previously reported from Iran only (Nicoletti and Zimowska, 2023). However, this species is spread worldwide as an endophyte of many plants (Nicoletti et al., 2021; Wang et al., 2021), and it is considered a valuable source of antimicrobial products (Ibrahim et al., 2024). One more species, *Melanconium hedericola* (Sordariomycetes, Diaporthales, Melanconiaceae), was also previously reported from Iran only (Nicoletti and Zimowska, 2023); no other mentions on its occurrence and ecological role could be found in the literature, likely in relation to its recent characterization (Crous et al., 2014).

Some other fungi represented in our sample, such as *Cytospora* (Diaporthales, Cytosporaceae)*, Diaporthe* (Diaporthales, Diaporthaceae) and *Alternaria* (Pleosporaceae), are known as pathogens of hazelnut (Wiman et al., 2019; Arciuolo et al., 2020; Waqas et al., 2024; Martino et al., 2025); this feature may imply that their recovery from asymptomatic tissues derives from interception during the latency period of disease cycle. Nevertheless, they are all known as producers of bioactive secondary metabolites (Kianfé et al., 2023; Wei et al., 2023; Zhao et al., 2023), which may have some relevance in contrasting bacterial infections in the field; by obvious reasons, such hypothesis cannot be further explored in the perspective of considering these fungi for applications in control strategies.

Finally, the occurrence of fungi belonging to the genera *Talaromyces* (Eurotiomycetes, Eurotiales, Trichocomaceae) and *Cladosporium* (Dothideomycetes, Cladosporiales, Cladosporiaceae) is noteworthy. Species of both these genera are widespread in every environment on Earth and reported as endophytic associates or to be involved in complex ecological relationships with plants, establishing tritrophic interactions with their pests and pathogens (Nicoletti and Becchimanzi, 2021; Nicoletti et al., 2022a, 2024; Pereira et al., 2024; Abbas et al., 2025). Moreover, they are known as producers of a plethora of bioactive compounds (Lei et al., 2022; Abdelatty et al., 2024; Salvatore et al., 2024). Contrary to what indicated by the closest ITS GenBank match, isolate HN10I was identified as *Talaromyces purpureogenus* based on its *rpb2* and *tub* sequences; although to the best of our knowledge this is the first report on hazelnut, this species has been previously mentioned as an endophytic associate on many plants (Nicoletti et al., 2020, 2022a; Hu et al., 2023). *Cladosporium* spp. are also described as widespread endophytic associates of plants, including hazelnut (Nicoletti and Zimowska, 2023). In our sample, there are two isolates of this genus (HN01C and HN05A) having *C. perangustum* and *C. cladosporioides* as the closest GenBank matches, respectively. The preliminary identification of the first isolate was later confirmed through sequencing *tef* as a more reliable molecular marker, while isolate HN05A was labeled as *C. cladosporioides* s.c.; in fact, the taxonomy of this species aggregate is still under verification and accurate phylogenetic assessments could be necessary for a correct classification (Becchimanzi et al., 2021).

Results of the antibacterial assays carried out against PA and XAC are reported in Table 2. After both bacterial strains had preliminarily demonstrated to grow well in PDB, the assays were performed by directly inoculating the filtered broth obtained from the fungal cultures.

**Table 2:**
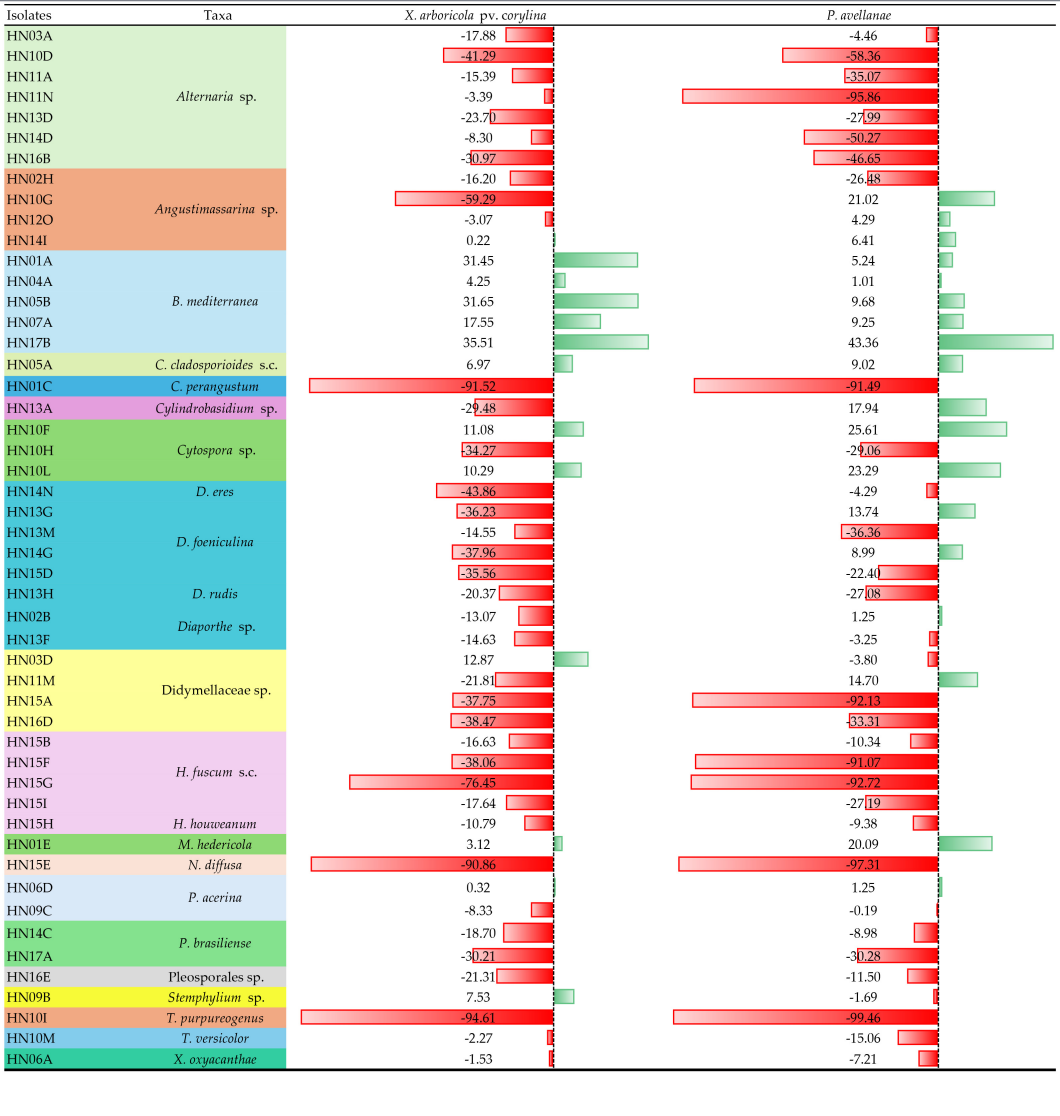
Effects on bacterial growth of culture filtrates of isolates of hazelnut endophytic fungi.

The reported values (%), calculated as means of three replicates, are representative of the growth inhibition (red bars) or promotion (green bars) in comparison to the untreated control.

The examination of data in Table 2 shows that a great proportion of the isolates (39) determined a reduction in the growth of the phytopathogenic bacteria in the PDB medium. The presence of a lesser number of promoter or neutral strains is not surprising, considering that, in the complexity of the interspecific ecological relationships, pathogens may in turn be adapted to overcome or to take advantage by the concomitant occurrence of some microbiome associates. At the same time, the reaction of the two bacterial strains was uneven, in line with the general outcome of research on antibiotics that these effects are not systematically expressed against any microbial species, and that the bacterial growth response is specific to each bacterial strain (Mitosch and Bollenbach, 2014).

**Figure 1:**
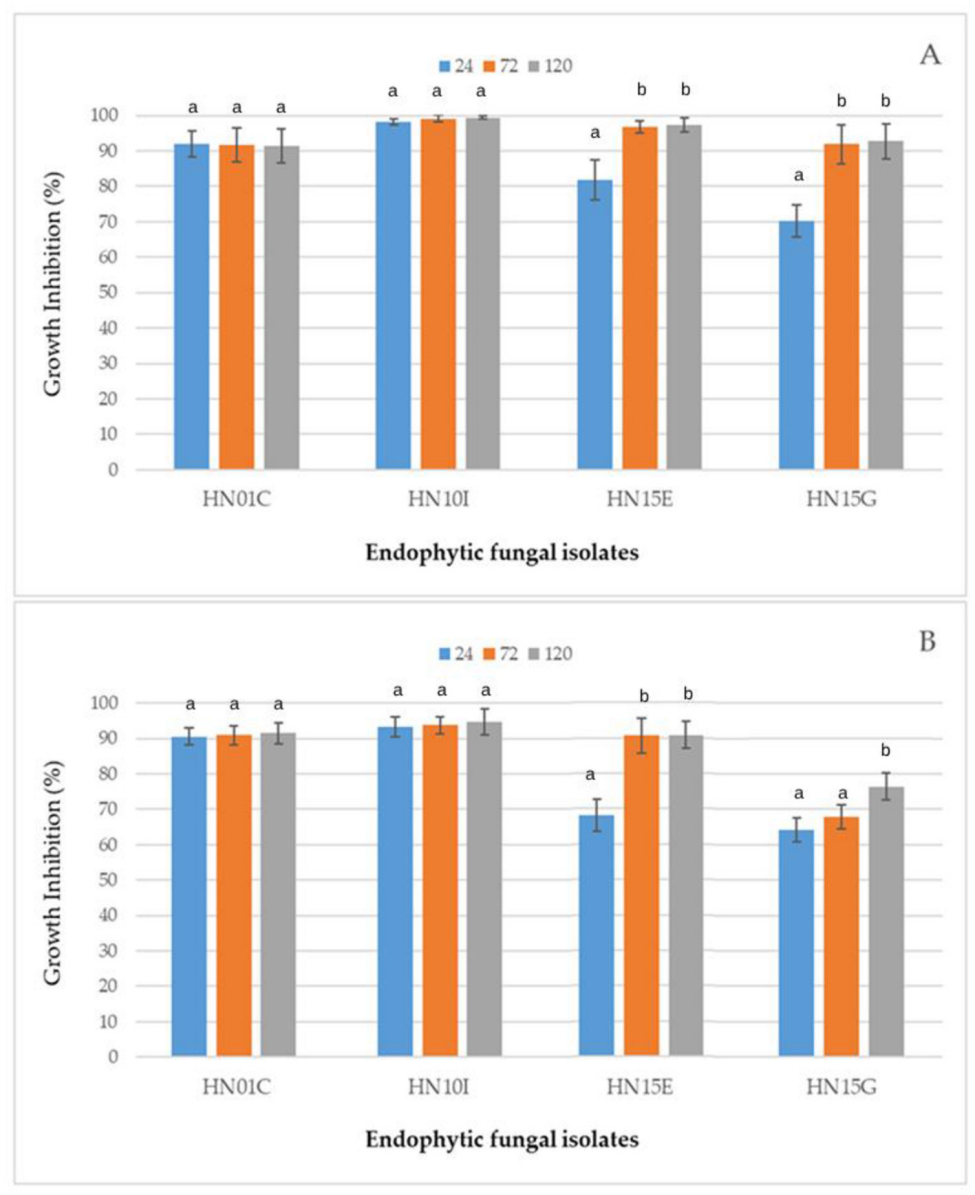
Growth inhibition of *P. avellanae* **(A)** and *X. arboricola* pv. *corylina* **(B)** as assessed at 24, 48, and 120 h of incubation in culture filtrates of four endophytic fungal isolates (HN01C, HN10I, HN15E, and HN15G). Values are expressed as mean ± standard deviation of three independent replicates (*n* = 3). Different letters above the bars indicate statistically significant differences for each isolate at the three incubation times, as determined by one-way ANOVA followed by Duncan's *post hoc* test (*p* ≤ 0.05).

In the context of a wide variation in the results of the assays, some coherent indications can be deduced for a few taxa which are represented by more than a single isolate in our sample. Particularly, all the five isolates of *B. mediterranea* promoted bacterial growth, while all the *Alternaria* and the *Hypoxylon* isolates, along with the two isolates of *P. brasiliense*, displayed inhibitory effects. Isolates of *Diaporthe* were also generally inhibitory, apart from a few cases producing scanty promoting effects toward PA, while an erratic response resulted for the heterogeneous groups of isolates belonging to the genera *Cytospora* and *Angustimassarina*, and the family Didymellaceae. A sharp inhibitory effect (above 50%) was induced by a limited number of isolates. This number was higher in the case of PA, which overall proved to be more sensitive than XAC. However, four isolates stood out for high inhibitory activity against both bacteria, namely HN01C, HN10I, HN15E, and HN15G. Their score was over 90% against both bacteria except for HN15G, whose activity was slightly less on XAC. The inhibitory effects by these isolates as recorded at 48, 72 and 120 hr post-inoculation are shown in Figure 1. It is interesting to note that for HN01C and HN10I the inhibition was over 90% since the first observation, while the two xylariaceous isolates HN15E and HN15G showed an increasing inhibitory trend over time on both bacteria.

## 4 Discussion

In an age when the need is arising to implement environmentally sound crop management practices, investigations on plant microbiomes provide a basic contribution to improving our understanding of the ecological conditions which shape crop production (Hao et al., 2024; Compant et al., 2025). As enabling a comparative examination of the species assortment and prevalence, the accumulation of data from various climatic and agricultural contexts is necessary to organize this information for its use and interpretation in the aim to promote plant health.

**Figure 2:**
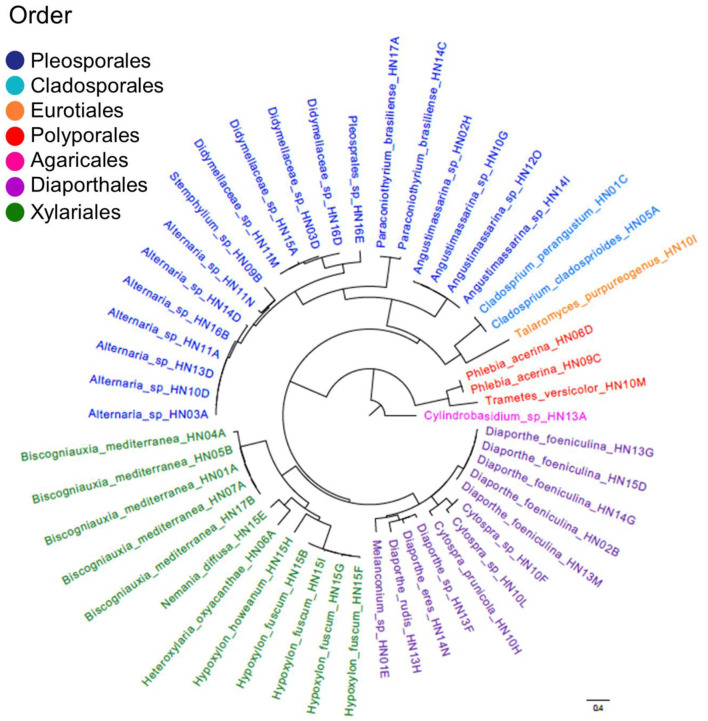
Dendrogram showing the diversity and the phylogenetic relationships among the hazelnut endophytic isolates considered in this study.

Besides an intrinsic descriptive significance in terms of biodiversity (Figure 2), our study confirmed that a panel of strains showing potential as biocontrol agents can be selected when starting from a wider set of isolates extracted from the plant microbiome, and that endophytic fungi represent a valuable source to be probed in this perspective. Indeed, their capacity to produce antimicrobial products has emerged in many studies carried out on a variety of crops (Nicoletti et al., 2020; Caruso et al., 2022). At the same time, inhibitory effects of microbial culture filtrates are commonly interpreted as dependent on their content in bioactive secondary metabolites (Izurdiaga et al., 2024); this inference stimulates further insights for the definition of protocols to assess if these fundamental properties are also exerted *in vivo*. With reference to hazelnut, a recent study is noteworthy reporting that endophytic strains belonging to the *Fusarium citricola* s.c. were able to produce enniatins *in planta*, supporting the conjecture that a role in defensive mutualism may be played by these cyclohexadepsipeptides (Zimowska et al., 2024). As in the case of enniatins, many fungal products have multiple effects against pathogens and pests (Grabka et al., 2022). Thus, the reported potential of the endophytic associates of hazelnut may also prompt further assessments on their role in contrasting the agents of other adversities, such as the stink bugs (Bosco et al., 2018), the many fungal pathogens affecting trunk (Martino et al., 2025), as well as those causing mycotoxin contamination of kernels (Salvatore et al., 2023; Sen and Kabak, 2025). As examined in the previous section, the most effective isolates all belong to species known to be able to synthesize antibacterial compounds, namely *C. perangustum, T. purpureogenus, N. diffusa* and *H. fuscum*, which support the inference that they could provide biochemical tools performing an active role in hazelnut defense. However, as the isolation of these fungi was done from a specific part of the sampled plants, the question arises whether they spread throughout the host organism in such a way to sistemically perform this function. Moreover, does bioactivity of their culture filtrates depend on a major compound or is the synergism of several metabolites needed to ensure the antibacterial effect? Can the biosynthetic aptitudes of different strains generate additive effects? Or, conversely, could they contrast one another when concomitantly present in the plant tissues? Indeed, all these questions require to be addressed in integrative studies to achieve the aim of identifying synthetic microbial communities which can effectively be exploited in plant protection (Shayanthan et al., 2022; Wang et al., 2023). In view of selecting the synthetic communities, such preliminary studies not only help to pick the species which better perform as candidate defensive mutualists, but also to exclude those which could rather be detrimental. In this respect, our findings clearly show that the secondary metabolism of *B. mediterranea* could eventually support the proliferation of the bacterial pathogens, with the possible onset of a synergism which might in turn amplify the pathogenic aptitude of this fungus.

In addition to the species of the four selected strains, the ecological role of *P. brasiliense* also deserves more accurate consideration. Indeed, its frequent occurrence as a component of hazelnut mycobiome should be examined considering the recent finding that it can induce production of paclitaxel (taxol) in *C. avellana* cell cultures (Salehi et al., 2019). In fact, this blockbuster antitumor drug also possesses antimicrobial properties (Pandy et al., 2023); thus, the eventual demonstration of the promotion of its biosynthesis *in planta* would consistently support the concept that *P. brasiliense* is actively involved in defensive mutualism by inducing the release of this compound, and possibly other bioactive secondary metabolites.

## 5 Conclusions

The antibacterial properties of a panel of 50 isolates of endophytic fungi recovered from asymptomatic hazelnut plants across wild and cropping contexts in Southern Italy were investigated using *in vitro* assays against two key pathogens of this crop, *P. avellanae* and *X. arboricola* pv. *corylina*. Four strains, taxonomically identified as *C. perangustum, N. diffusa, T. purpureogenus* and *H. fuscum* s.c., displayed the better inhibitory effects. Their ability to colonize hazelnut plants upon artificial inoculation, to release antimicrobial products *in vivo*, as well as their potential application in the control of bacterial diseases of hazelnut will be further investigated.

## Data Availability

The datasets presented in this study can be found in online repositories. The names of the repository/repositories and accession number(s) can be found in the article/supplementary material.

## References

[B1] Abbas A. Ali S. Mubeen M. Hussain A. Gutumsary K. A. Hussain B. . (2025). “*Talaromyces* spp. are promising biocontrol agents for sustainable agriculture”, in *Microbial Biocontrol Techniques: Importance in Ensuring Food Security*. eds. Kumar A. and Solanki M K. M.K (Singapore: Springer Nature) 245–280. doi: 10.1007/978-981-97-8739-5_13

[B2] Abdelatty N. A. Attia E. Z. Hamed A. N. E. Abdelmohsen U. R. Desoukey S. Y. Fouad M. A. . (2024). Recent updates on chemo biodiversity of the genus *Cladosporium*. Nat. Prod. J. 15:E22103155328257. doi: 10.2174/0122103155328257241021033118

[B3] Arciuolo R. Santos C. Soares C. Castello G. Spigolon N. Chiusa G. . (2020). Molecular characterization of *Diaporthe* species associated with hazelnut defects. Front. Plant Sci. 11:611655. doi: 10.3389/fpls.2020.611655 33362837 PMC7759530

[B4] Becchimanzi A. Zimowska B. Calandrelli M. M. De Masi L. Nicoletti R. (2025). Genome sequencing of a *Fusarium* endophytic isolate from hazelnut: phylogenetic and metabolomic implications. Int. J. Mol. Sci. 26:4377. doi: 10.3390/ijms26094377 40362614 PMC12072968

[B5] Becchimanzi A. Zimowska B. Nicoletti R. (2021). Cryptic diversity in *Cladosporium cladosporioides* resulting from sequence-based species delimitation analyses. Pathogens 10:1167. doi: 10.3390/pathogens10091167 34578199 PMC8472012

[B6] Becker K. Stadler M. (2021). Recent progress in biodiversity research on the Xylariales and their secondary metabolism. J. Antib. 74, 1–23. doi: 10.1038/s41429-020-00376-0 PMC7732752 33097836

[B7] Biagetti E. Pancino B. Martella A. La Porta I. M. Cicatiello C. Gregorio D. . (2023). Is hazelnut farming sustainable? an analysis in the specialized production area of Viterbo. Sustainability 15, 10702. doi: 10.3390/su151310702

[B8] Bosco L. Moraglio S. T. Tavella L. (2018). *Halyomorpha halys*, a serious threat for hazelnut in newly invaded areas. J. Pest Sci. 91, 661–670. doi: 10.1007/s10340-017-0937-x

[B9] Busby P. E. Ridout M. Newcombe G. (2016). Fungal endophytes: modifiers of plant disease. Plant Mol. Biol. 90, 645–655. doi: 10.1007/s11103-015-0412-0 26646287

[B10] Caruso D. J. Palombo E. A. Moulton S. E. Zaferanloo B. (2022). Exploring the promise of endophytic fungi: a review of novel antimicrobial compounds. Microorganisms 10:1990. doi: 10.3390/microorganisms10101990 36296265 PMC9607381

[B11] Compant S. Cassan F. Kostić T. Johnson L. Brader G. Trognitz F. . (2025). Harnessing the plant microbiome for sustainable crop production. Nature Rev. Microbiol. 23, 9–23. doi: 10.1038/s41579-024-01079-1 39147829

[B12] Costa D. Ramos V. Tavares R. M. Baptista P. Lino-Neto T. (2022). Phylogenetic analysis and genetic diversity of the xylariaceous ascomycete *Biscogniauxia mediterranea* from cork oak forests in different bioclimates. Sci. Rep. 12:2646. doi: 10.1038/s41598-022-06303-7 35173202 PMC8850622

[B13] Crous P. W. Wingfield M. J. Schumacher R. K. Summerell B. A. Giraldo A. Gené J. . (2014). Fungal planet description sheets. Persoonia 33, 212–289. doi: 10.3767/003158514X685680 25737601 PMC4312934

[B14] Demir Ö. Schmidt K. Schulz B. Stradal T. E. Surup F. (2025). Cytochalasins from the ash endophytic fungus *Nemania diffusa* DSM 116299. Molecules 30:957. doi: 10.3390/molecules30040957 40005267 PMC11858462

[B15] Dogan H. H. Öztürk C. Kaşik G. Aktaş S. (2005). A checklist of Aphyllophorales of Turkey. Pakistan J. Bot. 37, 459–485.

[B16] FAOStatistics (2025). Available online at: www.fao.org/statistics/en (Accessed December 10, 2025).

[B17] Franco M. E. Wisecaver J. H. Arnold A. E. Ju Y. M. Slot J. C. Ahrendt S. . (2022). Ecological generalism drives hyperdiversity of secondary metabolite gene clusters in xylarialean endophytes. New Phytol. 233, 1317–1330. doi: 10.1111/nph.17873 34797921

[B18] Fuso A. Risso D. Rosso G. Rosso F. Manini F. Manera I. . (2021). Potential valorization of hazelnut shells through extraction, purification and structural characterization of prebiotic compounds: a critical review. Foods 10:1197. doi: 10.3390/foods10061197 34073196 PMC8229101

[B19] Glass N. L. Donaldson G. C. (1995). Development of primer sets designed for use with the PCR to amplify conserved genes from filamentous ascomycetes. Appl. Environ. Microbiol. 6, 1323–1330. doi: 10.1128/aem.61.4.1323-1330.1995 PMC167388 7747954

[B20] Grabka R. d'Entremont T. W. Adams S. J. Walker A. K. Tanney J. B. Abbasi P. A. . (2022). Fungal endophytes and their role in agricultural plant protection against pests and pathogens. Plants 11:384. doi: 10.3390/plants11030384 35161365 PMC8840373

[B21] Guevara-Suarez M. Sutton D. A. Cano-Lira J. F. García D. Martin-Vicente A. Wiederhold N. . (2016). Identification and antifungal susceptibility of *Penicillium*-like fungi from clinical samples in the United States. J. Clin. Microbiol. 54, 2155–2161. doi: 10.1128/JCM.00960-16 27280422 PMC4963513

[B22] Hao J. R. Li Y. Ge Y. (2024). Harnessing the plant microbiome for environmental sustainability: from ecological foundations to novel applications. Sci. Total Environ. 951:175766. doi: 10.1016/j.scitotenv.2024.175766 39187075

[B23] Himani S. Krishnappa M. (2020). *Xylaria oxyacanthae* (Xylariaceae), a new record on *Diospyros melanoxylon* from India. Stud. Fungi 5, 485–490. doi: 10.5943/sif/5/1/29

[B24] Hu X. Saravanakumar K. Park S. Han K. S. Wang M. H. (2023). Isolation, characterization, antioxidant, and wound healing activities of extracellular polysaccharide from endophytic fungus *Talaromyces purpureogenus*. Appl. Biochem. Biotechnol. 195, 3822–3839. doi: 10.1007/s12010-022-04187-x 36260249

[B25] Ibrahim S. R. Alzain A. A. Elbadwi F. A. Koshak A. E. AlSaedi A. H. Ashour A. . (2024). Shedding light on *Paraconiothyrium brasiliense*: Secondary metabolites, biological activities, and computational studies. J. Appl. Pharm. Sci. 14, 35–52. doi: 10.7324/JAPS.2024, 184503.

[B26] Izurdiaga D. Sánchez-López Á. M. Fernández-San Millán A. Poveda J. (2024). Cell-free filtrates from plant pathogens: Potential new sources of bioactive molecules to improve plant health. Crop Prot. 176, 106477. doi: 10.1016/j.cropro.2023.106477

[B27] Kashanian A. Panjekeh N. Taliei F. (2021). Investigating diversity and spatial distribution of endophytic fungi in hazelnut (*Corylus avellana*) in its different habitats of Iran. Biol. J. Microorg. 10, 53–69. doi: 10.22108/bjm.2021.125722.1345

[B28] Kianfé B. Y. Tchamgoue J. Narmani A. Teponno R. B. Njouonkou A. L. Stadler M. . (2023). Bioactive secondary metabolites from fungi of the genus *Cytospora* Ehrenb. (Ascomycota). Molecules 28:3120. doi: 10.3390/molecules28073120 37049883 PMC10096137

[B29] Kim D. H. Kawg Y. N. Kim H. S. Han S. K. Kim C. S. Lee J. K. . (2025). Morphological and phylogenetic characterization of four additional *Xylaria*-associated species: three new species and one newly recognized species in Korea. Mycobiology 53, 747–759. doi: 10.1080/12298093.2025, 2551300. 40926781 PMC12416027

[B30] Krol E. Machowicz-Stefaniak Z. Zalewska E. (2004). Bacteria damaging the fruit of hazel (*Corylus avellana* L.) cultivated in South-East Poland. Acta Scientiarum Polonorum Hortorum Cultus 32, 75–84. doi: 10.24326/asphc.2004.2.9

[B31] Lambert C. Pourmoghaddam M. J. Cedeño-Sanchez M. Surup F. Khodaparast S. A. Krisai-Greilhuber I. . (2021). Resolution of the *Hypoxylon fuscum* complex (Hypoxylaceae, Xylariales) and discovery and biological characterization of two of its prominent secondary metabolites. J. Fungi 7:131. doi: 10.3390/jof7020131 PMC7916920 33670169

[B32] Lamichhane J. R. Varvaro L. (2014). *Xanthomonas arboricola* disease of hazelnut: current status and future perspectives for its management. Plant Pathol. 63, 243–254. doi: 10.1111/ppa.12152

[B33] Lei L. R. Gong L. Q. Jin M. Y. Wang R. Liu R. Gao J. . (2022). Research advances in the structures and biological activities of secondary metabolites from *Talaromyces*. Front. Microbiol. 13:984801. doi: 10.3389/fmicb.2022.984801 36060779 PMC9437523

[B34] Liu Y. J. Whelen S. Hall B. D. (1999). Phylogenetic relationships among ascomycetes: evidence from an RNA polymerase II subunit. Mol. Biol. 16, 1799–1808. doi: 10.1093/oxfordjournals.molbev.a026092 10605121

[B35] Ma H. Song Z. Pan X. Li Y. Yang Z. Qu Z. . (2022). Multi-gene phylogeny and taxonomy of *Hypoxylon* (Hypoxylaceae, Ascomycota) from China. Diversity 14:37. doi: 10.3390/d14010037

[B36] Martino I. Monchiero M. Gullino M. L. Guarnaccia V. (2025). Characterization and pathogenicity of fungal species associated with hazelnut trunk diseases in North-western Italy. J. Plant Pathol. 107, 87–105. doi: 10.1007/s42161-024-01595-2

[B37] Mitosch K. Bollenbach T. (2014). Bacterial responses to antibiotics and their combinations. Environ. Microbiol. Rep. 6, 545–557. doi: 10.1111/1758-2229.12190 25756107

[B38] Nera E. Paas W. Reidsma P. Paolini G. Antonioli F. Severini S. . (2020). Assessing the resilience and sustainability of a hazelnut farming system in central Italy with a participatory approach. Sustainability 12:343. doi: 10.3390/su12010343

[B39] Nguyen T. T. T. Frisvad J. C. Kirk P. M. Lim H. J. Lee H. B. (2021). Discovery and extrolite production of three new species of *Talaromyces* belonging to sections *Helici* and *Purpurei* from freshwater in Korea. J. Fungi 7:722. doi: 10.3390/jof7090722 PMC8471979 34575760

[B40] Nicoletti R. Andolfi A. Salvatore M. M. (2022a). “Endophytic Fungi of the Genus *Talaromyces* and Plant Health”. in, *Microbial Endophytes and Plant Growth*. eds. Solanki M.K. Yadav M.K. Singh B.P. and Gupta V.K. (Cambridge, MA: Academic Press) 183–213. doi: 10.1016/B978-0-323-90620-3, 00004–0.

[B41] Nicoletti R. Beccaro G. L. Sekara A. Cirillo C. Di Vaio C. (2021). Endophytic fungi and ecological fitness of chestnuts. Plants 10:542. doi: 10.3390/plants10030542 33805750 PMC7999096

[B42] Nicoletti R. Becchimanzi A. (2021). *Talaromyces*–insect relationships. Microorganisms 10:45. doi: 10.3390/microorganisms10010045 35056494 PMC8780841

[B43] Nicoletti R. Di Vaio C Cirillo C. C. (2020). Endophytic fungi of olive tree. Microorganisms 8:1321. doi: 10.3390/microorganisms8091321 32872625 PMC7565531

[B44] Nicoletti R. Petriccione M. Curci M. Scortichini M. (2022b). Hazelnut-associated bacteria and their implications in crop management. Horticulturae 8:1195. doi: 10.3390/horticulturae8121195

[B45] Nicoletti R. Russo E. Becchimanzi A. (2024). *Cladosporium*—insect relationships. J. Fungi 10:78. doi: 10.3390/jof10010078 PMC10820778 38276024

[B46] Nicoletti R. Zimowska B. (2023). Endophytic fungi of hazelnut (*Corylus avellana*). Plant Prot. Sci. 59, 107–123. doi: 10.17221/133/2022-PPS

[B47] Ordynets A. Savchenko A. Akulov A. Yurchenko E. Malysheva V. F. Kõljalg U. . (2017). Aphyllophoroid fungi in insular woodlands of eastern Ukraine. *Biodiv*. Data J. 5:e22426. doi: 10.3897/BDJ.5.e22426 PMC5769729 29362557

[B48] Pandy R. Kumar S. S. Suresh P. Annaraj J. Pandi M. Vellasamy S. . (2023). Screening and characterization of fungal taxol-producing endophytic fungi for evaluation of antimicrobial and anticancer activities. Open Chem. 21:20220344. doi: 10.1515/chem-2022-0344

[B49] Pereira C. M. Sarmiento S. S. Colmán A. A. Belachew-Bekele K. Evans H. C. Barreto R. W. . (2024). Mycodiversity in a micro-habitat: twelve *Cladosporium* species, including four new taxa, isolated from uredinia of coffee leaf rust, Hemileia vastatrix. Fungal Syst. Evol. 14, 9–33. doi: 10.3114/fuse.14, 02. 39830303 PMC11736085

[B50] Pereira L. B. Thomazella D. P. Teixeira P. J. (2023). Plant-microbiome crosstalk and disease development. Curr. Opin. Plant Biol. 72:102351. doi: 10.1016/j.pbi.2023.102351 36848753

[B51] Pergola M. Maffia A. Picone A. Palese A. M. Altieri G. Celano G. . (2024). Hazelnut cultivation in the campania region: environmental sustainability of the recovery of pruning residues and shells through the life cycle assessment methodology. Sustainability 16:7533. doi: 10.3390/su16177533

[B52] Pi Y. H. Long S. H. Wu Y. P. Liu L. L. Lin Y. Long Q. D. . (2021). A taxonomic study of *Nemania* from China, with six new species. MycoKeys 83, 39–67. doi: 10.3897/mycokeys.83.69906 34539206 PMC8408098

[B53] Pulawska J. Kaluzna M. Kolodziejska A. Sobiczewski P. (2010). Identification and characterization of *Xanthomonas arboricola* pv. *corylina* causing bacterial blight of hazelnut: a new disease in Poland. J. Plant Pathol. 92, 803–806. doi: 10.4454/JPP.V92I3.331

[B54] Rabiey M. Hailey L. E. Roy S. R. Grenz K. Al-Zadjali M. A. Barrett G. A. . (2019). Endophytes vs tree pathogens and pests: can they be used as biological control agents to improve tree health? Eur. J. Plant Pathol. 155, 711–729. doi: 10.1007/s10658-019-01814-y

[B55] Romero-Aroca A. Rovira M. Cristofori V. Silvestri C. (2021). Hazelnut kernel size and industrial aptitude. Agriculture 11:1115. doi: 10.3390/agriculture11111115

[B56] Salehi M. Moieni A. Safaie N. Farhadi S. (2019). Elicitors derived from endophytic fungi *Chaetomium globosum* and *Paraconiothyrium brasiliense* enhance paclitaxel production in *Corylus avellana* cell suspension culture. Plant Cell Tissue Organ Cult. 136, 161–171. doi: 10.1007/s11240-018-1503-9

[B57] Salvatore M. M. Andolfi A. Nicoletti R. (2023). Mycotoxin contamination in hazelnut: current status, analytical strategies, and future prospects. Toxins. 15:99. doi: 10.3390/toxins15020099 36828414 PMC9965003

[B58] Salvatore M. M. Nicoletti R. Fiorito F. Andolfi A. (2024). Penicillides from *Penicillium* and *Talaromyces*: chemical structures, occurrence and bioactivities. Molecules 29:3888. doi: 10.3390/molecules29163888 39202967 PMC11356976

[B59] Scortichini M. Marcelletti S. Ferrante P. Firrao G. (2013). A genomic redefinition of *Pseudomonas avellanae* species. PLoS One 8:e75794. doi: 10.1371/journal.pone.0075794 24086635 PMC3783423

[B60] Scortichini M. Marchesi U. Rossi M. P. Prospero D.i. P . (2002a). Bacteria associated with hazelnut (*Corylus avellana* L.) decline are of two groups: *Pseudomonas avellanae* and strains resembling P. syringae pv. syringae. Appl. Environ. Microbiol. 68, 476–484. doi: 10.1128/AEM.68.2.476-484.2002 11823181 PMC126672

[B61] Scortichini M. Rossi M. P. Marchesi U. (2002b). Genetic, phenotypic and pathogenic diversity of *Xanthomonas arboricola* pv. *corylina* strains question the representative nature of the type strain. Front. Plant Sci. 14:1254107. doi: 10.3389/fpls.2023.1254107

[B62] Sen L. Kabak B. (2025). Mycotoxin contamination in hazelnuts: food safety challenges in a changing climate. J. Food Compos. Anal. 144:107699. doi: 10.1016/j.jfca.2025.107699

[B63] Shayanthan A. Ordoñez P. A. C. Oresnik I. J. (2022). The role of synthetic microbial communities (SynCom) in sustainable agriculture. Front. Agron. 4:896307. doi: 10.3389/fagro.2022.896307

[B64] Stadler M. Fournier J. Quang D. N. Akulov A. Y. (2007). Metabolomic studies on the chemical ecology of the Xylariaceae (Ascomycota). Nat. Prod. Commun. 2, 287–304. doi: 10.1177/1934578X0700200311

[B65] Tamm L. Thuerig B. Apostolov S. Blogg H. Borgo E. Corneo P. E. . (2022). Use of copper-based fungicides in organic agriculture in twelve European countries. Agronomy 12:673. doi: 10.3390/agronomy12030673

[B66] Thambugala K. M. Hyde K. D. Tanaka K. Tian Q. Wanasinghe D. N. Ariyawansa H. A. . (2015). Towards a natural classification and backbone tree for Lophiostomataceae, Floricolaceae, and Amorosiaceae fam. nov. Fungal Divers. 74, 199–266. doi: 10.1007/s13225-015-0348-3

[B67] Tomsovský M. Kolarik M. Pazoutová S. Homolka L. (2006). Molecular phylogeny of European *Trametes* (Basidiomycetes, Polyporales) species based on LSU and ITS (nrDNA) sequences. Nova Hedwigia 82, 269–280. doi: 10.1127/0029-5035/2006/0082-0269

[B68] Varvaro L. Fabi A. Magro P. Paparatti B. (2011). Aspetti fitosanitari della corilicoltura nel Viterbese. Corylus Co. 1, 21–37.

[B69] Vinci A. Lena B. Di. Portarena S. Farinelli D. (2023). Trend analysis of different climate parameters and watering requirements for hazelnut in Central Italy related to climate change. Horticulturae 9:593. doi: 10.3390/horticulturae9050593

[B70] Vocadlova K. Lüddecke T. Patras M. A. Marner M. Hartwig C. Benes K. . (2023). Extracts of *Talaromyces purpureogenus* strains from *Apis mellifera* bee bread inhibit the growth of *Paenibacillus* spp. in vitro. Microorganisms 11:2067. doi: 10.3390/microorganisms11082067 37630627 PMC10459140

[B71] Wang J. Shao S. Liu C. Song Z. Liu S. Wu S. . (2021). The genus *Paraconiothyrium*: species concepts, biological functions, and secondary metabolites. Crit. Rev. Microbiol. 47, 781–810. doi: 10.1080/1040841X.2021.1933898 34214001

[B72] Wang Z. Hu X. Solanki M. K. Pang F. (2023). A synthetic microbial community of plant core microbiome can be a potential biocontrol tool. J. Agric. Food Chem. 71, 5030–5041. doi: 10.1021/acs.jafc.2c08017 36946724

[B73] Waqas M. Guarnaccia V. Bardella S. Spadaro D. (2024). Molecular characterization and pathogenicity of *Diaporthe* species causing nut rot of hazelnut in Italy. Plant Dis. 108, 1005–1013. doi: 10.1094/PDIS-01-23-0168-RE 37883635

[B74] Wei W. Khan B. Dai Q. Lin J. Kang L. Rajput N. . (2023). Potential of secondary metabolites of *Diaporthe* species associated with terrestrial and marine origins. J. Fungi 9:453. doi: 10.3390/jof9040453 PMC10143158 37108907

[B75] White T. J. Bruns T. Lee S. Taylor J. W. (1990). “Amplification and Direct Sequencing of Fungal Ribosomal RNA Genes for Phylogenetics”, in PCR Protocols: A Guide to Methods and Applications eds. Innis M.A. Gelfand D.H. Sninsky J.J. White T.J. (New York, NY: Academic Press Inc.). 315–322. doi: 10.1016/B978-0-12-372180-8, 50042–1.

[B76] Wiman N. G. Webber J. B. III. Wiseman M. Merlet L. (2019). Identity and pathogenicity of some fungi associated with hazelnut (*Corylus avellana* L.) trunk cankers in Oregon. PLoS One 14:e0223500. doi: 10.1371/journal.pone.0223500 31600302 PMC6786572

[B77] Yildirim Ç. Bozoglu M. Urago G. G. (2024). Türkiye's competitive power in the world hazelnut market. Appl. Fruit Sci. 66, 921–928. doi: 10.1007/s10341-024-01053-4

[B78] Zhao S. Li J. Liu J. Xiao S. Yang S. Mei J. . (2023). Secondary metabolites of *Alternaria*: a comprehensive review of chemical diversity and pharmacological properties. Front. Microbiol. 13:1085666. doi: 10.3389/fmicb.2022.1085666 36687635 PMC9852848

[B79] Zíbarová L. Kout J. (2017). Xylariaceous pyrenomycetes from bohemia: species of *Biscogniauxia* and *Hypoxylon* new to the Czech Republic, and notes on other rare species. Czech Mycol. 69, 77–108. doi: 10.33585/cmy.69106

[B80] Zimowska B. (2021). “Taxonomical evaluation of Phoma: History of classification, current status and future directions”. in Phoma: Diversity, Taxonomy, Bioactivities, and Nanotechnology. eds. Rai M. Zimowska B. and Kövics G.J. (Cham, Switzerland: Springer) 13–34. doi: 10.1007/978-3-030-81218-8_2

[B81] Zimowska B. Ludwiczuk A. Manganiello G. Wojtanowski K. Kot I. Staropoli A. . (2024). *Fusarium* and hazelnut: a story of twists and turns. Agriculture 14:1080. doi: 10.3390/agriculture14071080

[B82] Zinnanti C. Schimmenti E. Borsellino V. Paolini G. Severini S. (2019). Economic performance and risk of farming systems specialized in perennial crops: an analysis of Italian hazelnut production. Agric. Syst. 176:102645. doi: 10.1016/j.agsy.2019.102645

